# Unveiling the impact of 16S rRNA gene intergenomic variation on primer design and gut microbiome profiling

**DOI:** 10.3389/fmicb.2025.1573920

**Published:** 2025-05-02

**Authors:** Sirinthorn Sunthornthummas, Rujipat Wasitthankasem, Pimonwan Phokhaphan, Nirinya Sudtachat, Alisa Wilantho, Chumpol Ngamphiw, Wanwisa Chareanchim, Sissades Tongsima

**Affiliations:** National Biobank of Thailand (NBT), National Center for Genetic Engineering and Biotechnology (BIOTEC), National Center for Genetic Engineering and Biotechnology, National Science and Technology Development Agency (NSTDA), Pathum Thani, Thailand

**Keywords:** 16S rRNA gene, gut microbiome profiling, primer design, intergenomic variation, microbial community

## Abstract

The 16S rRNA gene is crucial for bacterial identification, but primer biases and intergenomic variation can compromise its effectiveness, especially in complex ecosystems like the human gut microbiome. This study systematically evaluates 57 commonly used 16S rRNA primer sets through *in silico* PCR simulations against the SILVA database. We identified three promising primer sets (V3_P3, V3_P7, and V4_P10) that offer balanced coverage and specificity across 20 key genera of the core gut microbiome. Our findings reveal: (1) significant limitations in widely used “universal” primers, often failing to capture microbial diversity due to unexpected variability in conserved regions, (2) substantial intergenomic variation, even within traditionally conserved regions of the 16S rRNA gene, as demonstrated by Shannon entropy analysis, and (3) discrepancies between intergenomic patterns in NCBI and SILVA databases, highlighting the impact of database choices on taxonomic classification. These results challenge assumptions about 16S rRNA gene conservation and emphasize the need for tailored primer design informed by comprehensive sequence databases. We advocate for a multi-primer strategy to improve coverage and mitigate biases, ultimately enhancing the accuracy and reliability of gut microbiome profiling. This approach has potential applications beyond gut microbiome studies, including animal microbiome research and probiotic community profiling.

## Introduction

The human gut microbiome, a complex ecosystem of trillions of microorganisms, plays an important role in human health and disease. The microbiome interactions with the host’s physiology, immune system, and metabolism are important in various conditions, from gastrointestinal disorders to obesity, diabetes, and mental health disorders (Afzaal et al., 2022). Recent advances in molecular techniques have enabled the characterization of microbial compositions, including those within the human gut. Among these, 16S rRNA gene sequencing has become a cornerstone of bacterial and archaeal community research due to its cost-effectiveness, scalability, and ability to generate taxonomic profiles (Gao et al., 2021). The 16S rRNA gene, spanning approximately 1,500 nucleotides, is a conserved component of microbial ribosomes, containing both highly conserved and variable regions (Gao et al., 2021). Through analysis of substitution rates across the rRNA gene, nine hypervariable regions (V1–V9) have been identified, interspersed with 10 highly conserved regions. The conserved regions are typically targeted for primer design in PCR amplification, while the variable regions serve as molecular markers for bacterial taxonomic classification (Yarza et al., 2014; Pan et al., 2023).

Despite widespread use, 16S rRNA gene sequencing faces several challenges. A primary issue is amplification bias, which arises from variability in primer binding sites across diverse bacterial taxa (Johnson et al., 2019). This bias can result in suboptimal primer performance, particularly in capturing the full spectrum of microbial diversity. Such limitations are particularly evident in the inability of universal primers to adequately represent dominant, yet unculturable, bacteria in complex microbiome communities (Hofer, 2018). This problem likely stems from primers being designed based on limited datasets, primarily derived from culturable bacteria that may not fully reflect the diversity in modern microbiome studies.

Further complicating microbiome analysis is the choice of target regions within the 16S rRNA gene. The variable regions used for taxonomic classification significantly impact primer specificity, amplification efficiency, and the resolution power of taxonomic identification, all of which directly affect the accuracy of microbiome profiling (Pan et al., 2023; Johnson et al., 2019). For example, a urine microbiome study highlight discrepancies in microbial diversity estimates when comparing the V3–V4 and the V4–V5 target regions, demonstrating how target region selection can lead to inconsistent results (Heidrich et al., 2022).

The human microbiome project, initiated in the past decade, has significantly advanced our understanding of the dynamic and diverse bacterial communities in the human gut (Turnbaugh et al., 2007). This progress is largely due to the discovery of previously unculturable bacteria, made possible by high-throughput sequencing techniques (Hug, 2018). The growing dataset from these unculturable bacterial sources highlights the ongoing evolution of bacterial communities, which may cause shifts in the intergenomic patterns of the 16S rRNA gene. These shifts present new challenges for the application of 16S rRNA gene sequencing, particularly in accurately assessing microbial diversity. Notably, such shifts could affect both the conserved and variable regions of the gene, raising concerns about the accuracy of current primers used for gut microbiome profiling (Kitahara et al., 2012).

In addition to primer selection, the choice of reference database for taxonomic assignment plays a crucial role in microbiome studies (Balvočiūtė and Huson, 2017). Several databases, including GSR-DB (Molano et al., 2024), MIMt (Cabezas et al., 2024), GTDB (Parks et al., 2022), Greengenes (McDonald et al., 2012), SILVA (Quast et al., 2013), RDP (Maidak et al., 2001), and NCBI (Federhen, 2012) differ in their sequence curation, taxonomic hierarchies, and nomenclature. These differences can lead to discrepancies in species identification and hinder consistency across studies. The SILVA database, which includes Bacteria, Archaea, and Eukaryota, is curated through phylogenetic analysis of small subunit rRNAs (16S and 18S) using the SINA alignment tool (Quast et al., 2013). In contrast, the NCBI database, the largest repository of sequences, relies on taxonomic assignments provided by sequence submitters, with curated data limited to those in the RefSeq collection (Schoch et al., 2020).

**Figure 1:**
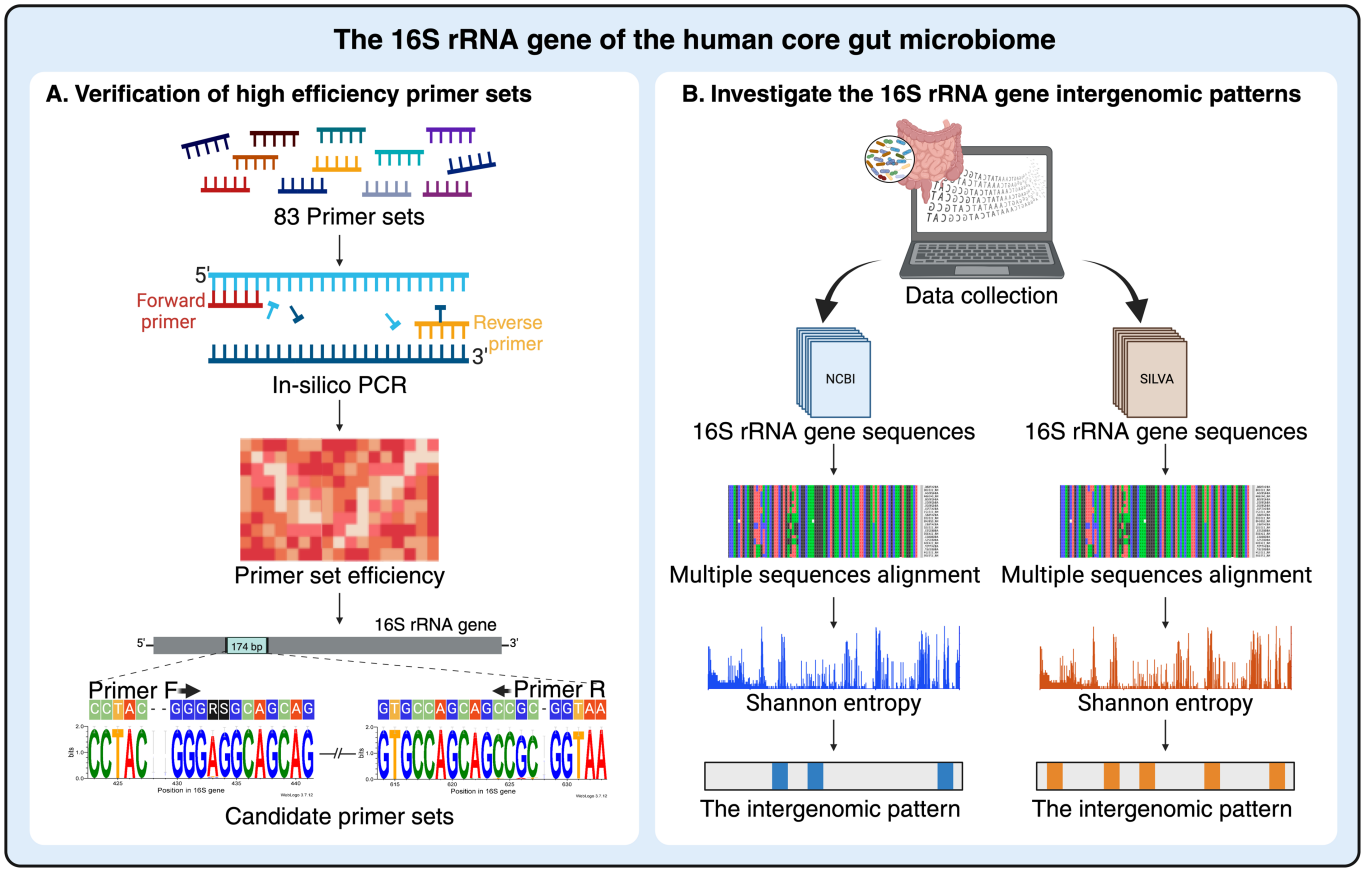
Schematic diagram of 16S rRNA gene analysis based on human core gut microbiome in this study. **(A)** Universal primer sets used in previous human microbiome studies were evaluated. The efficiency of these primers on the gut microbiota was re-assessed using an *in-silico* PCR approach to identify the most promising primer set for analyzing the core gut microbiome profiles. **(B)** The diversity of intergenomic patterns within 16S rRNA genes from human gut microbiota was examined to identify variations in both conserved and variable regions. The 16S rRNA gene sequences of the core gut microbiome were initially downloaded separately from the NCBI and SILVA databases, and each dataset was further analyzed for the intergenomic patterns using multiple sequence alignments and Shannon entropy graph analysis. Figure created in BioRender.com.

Despite widespread use of 16S rRNA gene sequencing for microbiome profiling, the impact of intergenomic variation on primer performance remains incompletely understood, particularly in complex environments like the human gut. This study addresses this knowledge gap by conducting a comprehensive *in silico* analysis of the core gut microbiome to evaluate the performance of universal 16S rRNA primers and characterize intergenomic variation patterns (Figure 1). Our findings provide valuable insights for optimizing primer design and enhancing the accuracy of future microbiome studies.

## Materials and methods

### Selection and curation of 16S rRNA primer sequences for gut microbiome analysis

To investigate the performance of 16S rRNA primer sets in profiling the human gut microbiome, we conducted a systematic review and compiled a comprehensive list of commonly used primers. PubMed^1^ was searched in June 2022 with the keywords “primer,” “16S,” “amplicon-based” and “human gut microbiome.” From 70 initial articles, 12 were selected based on: (1) publication date between 2012 and 2022, (2) Q1 journal or impact factor ≥3, (3) focus on human microbiome studies, and (4) evidence of primer assessment via *in silico* analysis or laboratory validation. Commercially available primer sets from Omega Bio-Services^2^ were also included. All primer sequences and sources are listed in Supplementary Table S1.

### *In silico* primer validation and selection of high-coverage candidates

Eighty-three primer pairs were initially compiled; 26 were removed due to identical forward and reverse sequences, leaving 57 unique pairs, targeting different 16S rRNA variable regions (V1–V9). A unique identifier was assigned to each pair based on its targeted region (e.g., V1_P1, V1_P2, etc.) as detailed in Supplementary Table S2. TestPrime 1.0^3^ (Klindworth et al., 2013) was then used to assess *in silico* performance of each primer pair against the SILVA SSU Ref NR 16S rRNA gene database (release 138.1), which contains 510,495 sequences (>1,200 bp for Bacteria/Eukaryota, >900 bp for Archaea). We applied a criterion of perfect alignment within primer degeneracy, meaning that matches were accepted if they aligned perfectly with any possible sequence within the degenerate primer pool. No mismatches were allowed outside of the designed degenerate positions. This analysis focused on the four dominant gut phyla (Actinobacteriota, Bacteroidota, Firmicutes, and Proteobacteria) (Turnbaugh et al., 2007; Cimadamore et al., 2019). Primer coverage was defined as the percentage of eligible sequences that were successfully amplified. Following Klindworth et al. (2013), primer pairs achieving ≥70% coverage across all four phyla were selected for further analysis. Among these, those that also achieved ≥90% coverage for at least four out of 20 representative genera were considered candidate primer sets for the gut microbiome, ensuring both broad phylum-level coverage and robust genus-level representation.

### Primer assessment using a mock gut microbiome community

To validate candidate primers under more complex conditions, we leveraged the ZymoBIOMICS^®^ Gut Microbiome Standard D6331 (Zymo Research, Irvine, CA). The dataset comprised 110 total 16S rRNA gene sequences derived from the 19 bacterial and archaeal strains present in the standard (after yeast exclusion; Supplementary Table S3). This higher number of sequences is due to multiple 16S rRNA gene copies per strain as reflect the true variation of these copies. Alignments were performed with MAFFT version 7 (Katoh et al., 2019), and sequence logos generated using WebLogo 3.^4^ Twenty representative core gut genera (Turnbaugh et al., 2007; Cimadamore et al., 2019) were examined for 16S rRNA gene variability, with reference sequences retrieved in May 2023 from both the NCBI^5^ and SILVA databases (see text footnote 3) (Quast et al., 2013). Sequences from NCBI were prioritized from the 16S Ribosomal RNA RefSeq Targeted Loci Project, whereas those from SILVA were drawn from SSU release 138.1.

All sequences underwent quality check via pairwise alignment against the conserved marker of 5′ end of the 16S rRNA gene, 5′-AGAGTTTGATCATGGCTCAG-3′, which used to define the 16S rRNA sub-regions (Yang et al., 2016), and only those aligning from the first position of the conserved region were retained. One hundred sequences per genus were randomly selected for further analysis, then aligned with MAFFT version 7 (Katoh et al., 2019) to produce multiple sequence alignments (MSAs). These alignments were trimmed to 1,500 bp, with position 1 corresponding to the start of conserved region 1 of *E. coli* strain 97–3,250. Shannon entropy values were computing using Entropy Plotter^6^ and analyzed in BioEdit (Hall, 1999). Regions with entropy >0.5 were classified as variable.

### Evaluating primer binding in the context of intergenomic variation

The binding specificity of candidate primer sets was examined in light of the observed intergenomic variation. For each genus, consensus sequences were derived from MSAs of NCBI and SILVA data. Candidate primers were then aligned to these consensus sequences to pinpoint binding sites. Sequence logos, generated by WebLogo3, highlighted nucleotide frequency distributions at each primer position, allowing comparisons between primer binding-site variability and overall intergenomic diversity. By mapping binding-site positions against the entropy results, we identified both conserved and variable segments and assessed how these patterns could affect primer performance.

## Results

### *In silico* performance of 16S rRNA primers across core gut microbiome taxa

We initiated our evaluation with 57 unique primer sets commonly used in gut microbiome studies, the majority (49/57, 85.97%) targeting multiple variable regions of the 16S rRNA gene, primarily V1–V4. Through a stringent *in silico* evaluation TestPrime 1.0 revealed that only 24 primer sets achieved our pre-defined coverage criterion of ≥ 70% across the four dominant core bacterial phyla (Supplementary Table S4). Most of qualified primer sets targeted the V3–V6 regions (15/24), corresponding to approximately nucleotide 300–1,100 of the 16S rRNA gene.

**Figure 2:**
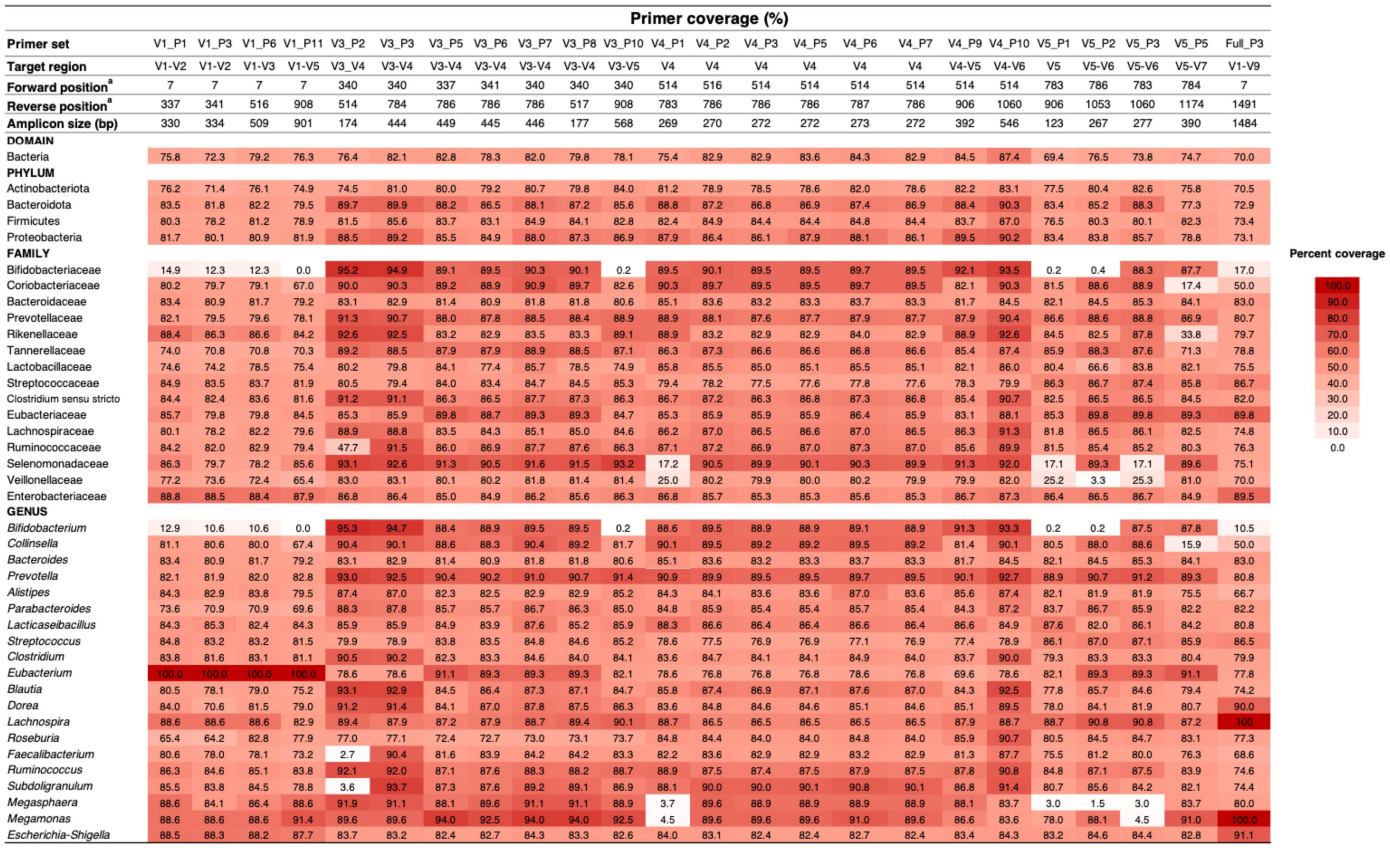
Primer efficiency for gut microbiome study. Primer coverage was evaluated using *in silico* PCR via the TestPrime 1.0 online tool. A heatmap illustrates the efficiency of primer pairs in amplifying the core gut microbiota. The heatmap organizes primer sets based on the region targeted by the forward primer, aligning bacterial sequences according to bacterial domain, phylum, family, and genus using SILVA taxonomy annotation. Primer sets are categorized into four groups based on their target regions: upstream (V1–V5), midstream (V3–V7), downstream (V5–V7), and full length (V1–V9). Resulting in amplicon sizes ranging from 124 to 1,484 base pairs. A color scale denotes primer efficiency, with darker shades indicating high amplification performance and white indicating an inability to amplify members of a bacterial genus.

Further *in silico* evaluation at family and genus levels revealed performance variations (Figure 2). While all 24 primer sets performed well at the phylum level, only 12 maintained ≥70% coverage across the core gut microbiome families and genera. Notably, we observed taxon-specific biases. Primers targeting the V1 region, while effective for *Eubacterium*, underperformed for *Bifidobacterium*. Primer sets targeting middle regions show reduced coverage for genera including *Faecalibacterium*, *Subdoligranulum*, *Bifidobacterium*, *Megasphaera*, and *Megamonas*. Similarly, primers targeting downstream regions (V5 and beyond) exhibited lower coverage for *Bifidobacterium*, *Collinsella*, *Megasphaera*, and *Megamonas*. Even the full-length primer set (Full_P3) failed to effectively capture *Bifidobacterium* and *Collinsella*.

Based on these *in silico* analyses, we ultimately selected three primer sets (V3_P3, V3_P7, and V4_P10) as promising candidates. These demonstrated broad coverage (V3–V7 regions; ~174–546 bp amplicon size) and minimal taxon-specific biases across diverse core gut microbiome taxa (Supplementary Table S5).

### Intergenomic variation of the 16S rRNA gene and implications for primer binding and performance

To understand the challenges and opportunities presented by 16S rRNA gene diversity for gut microbiome profiling, we characterized intergenomic variation across 20 core genera and evaluated the performance of three promising primer sets (V3_P3, V3_P7, and V4_P10) *in silico*. We analyzed 16S rRNA gene sequences from both NCBI and SILVA databases (Table 1). Entropy plots (Supplementary Figures S1, S2) revealed that most genera exhibited similar variation patterns between databases, with comparable entropy indices and overlapping regions of high sequence diversity. However, some genera showed database-specific conservation patterns (e.g., *Lacticaseibacillus* and *Eubacterium* were highly conserved in SILVA).

**Figure 3:**
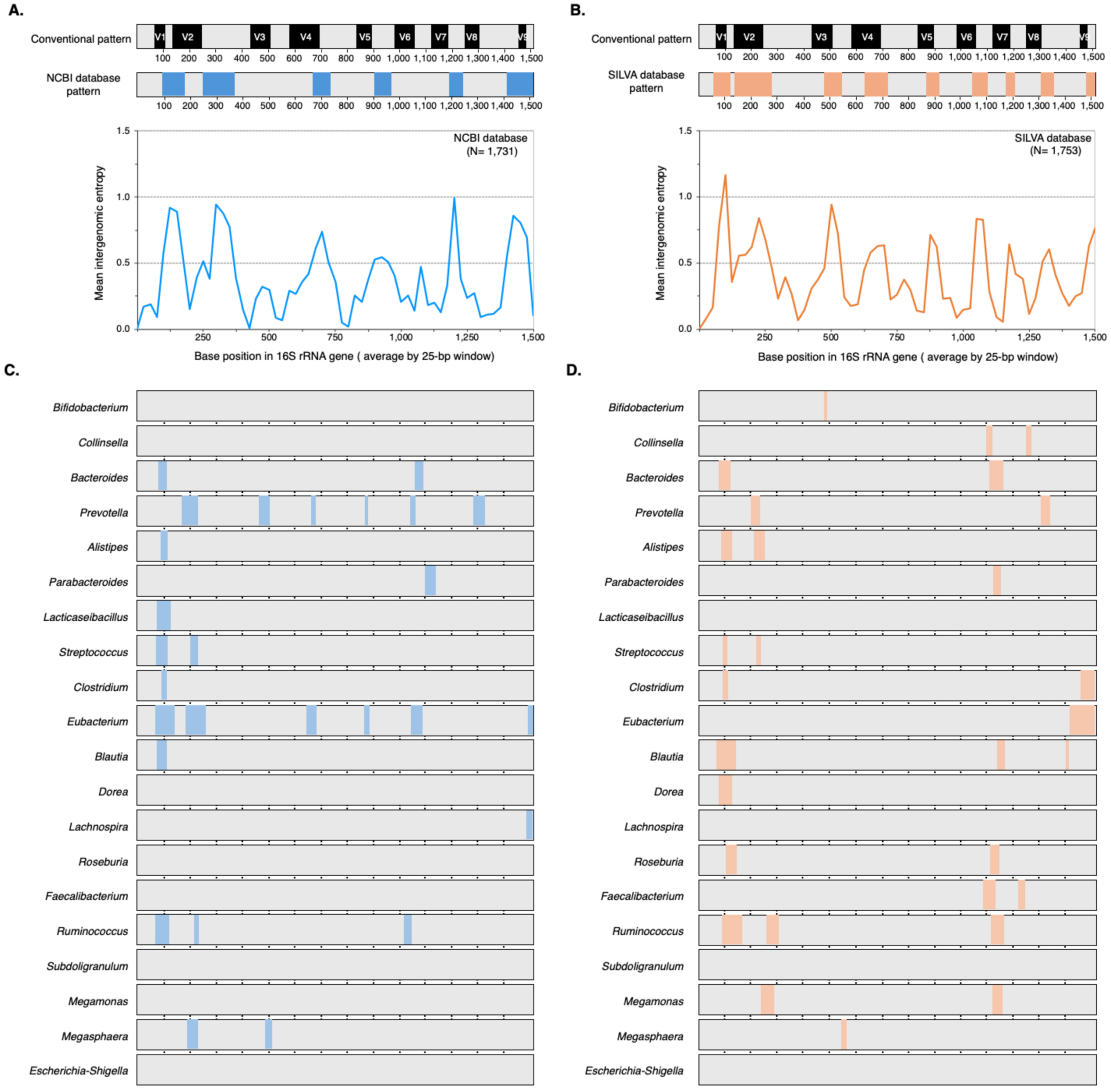
Intergenomic patterns of 16S rRNA genes within core human gut microbiome genera from NCBI and SILVA databases. **(A,B)** Comparison of the conventional 16S rRNA gene structure with intergenomic patterns derived from NCBI and SILVA databases, respectively. The upper panel displays the intergenomic pattern generated based on the mean entropy graph shown in the lower panel. **(C,D)** Intergenomic patterns at the genus level for sequences obtained from NCBI and SILVA, respectively. Variable regions, identified by a high entropy index (2 ≥ 0.5), are highlighted by colored boxes in all panels. Conserved regions were indicated in gray. Figure created in BioRender.com.

**Figure 4:**
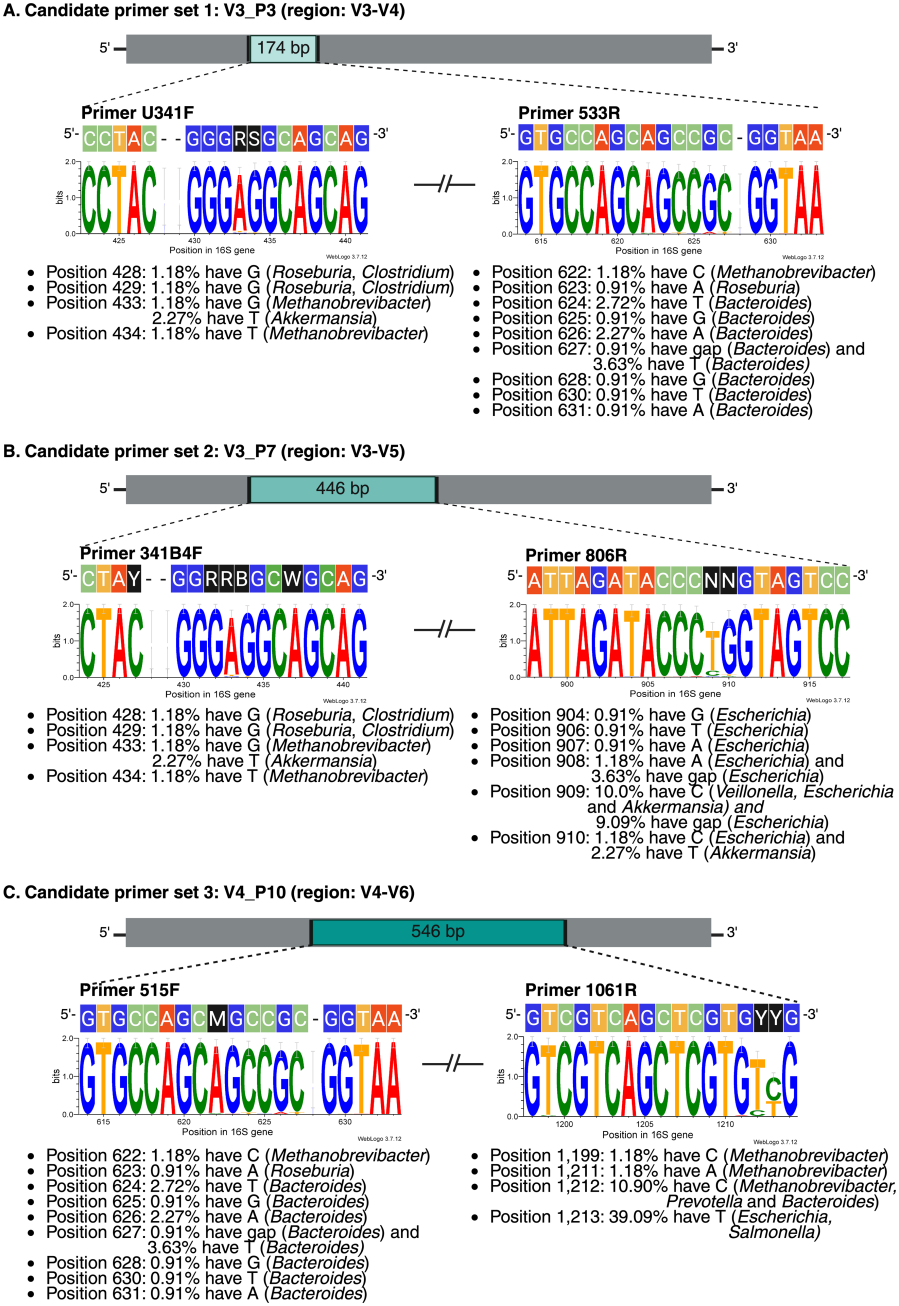
Primer binding site conservation in the high-complexity microbiome for three candidate primer sets: **(A)** V3_P3 (primers U341F and 533R), **(B)** V3_P7 (primers 341B4F and 806R), and **(C)** V4_P10 (primers 515F and 1061R). Each panel displays the forward and reverse primer sequences above their corresponding sequence logo. The sequence logos illustrate nucleotide conservation within the primer binding sites across 110 gut microbiome sequences. In this analysis, the reverse primer sequences were already reverse complemented, so the primer sequences are arranged from left to right, with the 3′ end on the right. The height of each letter represents the relative nucleotide frequency at each position, measured in bits and ranging from 0 (equal probability for all four nucleotides) to 2 (perfect conservation of the position). Genera with limitations for each primer set are noted below the sequence logo. Figure created in BioRender.com.

Comparison with the *E. coli* conventional 16S rRNA gene pattern (Yarza et al., 2014) showed that none of the 20 genera exhibited the full complement of nine variable regions in either NCBI or SILVA datasets (Figures 3C,D). Both databases showed comparable overall diversity, but NCBI exhibited greater variation in six genera (particularly *Prevotella* and *Eubacterium*), while SILVA showed greater variation in nine genera. The positions and sizes of the variable regions also deviated from the *E. coli* reference, with NCBI showing six moderately variable regions (Figure 3A) and SILVA showing nine more highly variable regions (Figure 3B). The target regions of the selected primers are shown in Supplementary Figure S3. V3_P3 is nested within the broader target region of V3_P7, while the 3′ end of the V3_P7 amplicon overlaps with the 5′ end of the V4_P10 amplicon. We then evaluated the candidate primers against a mock microbial community of 110 16S rRNA gene sequences from 19 representative gut microbiome taxa. Multiple sequence alignments of the primer binding sites are shown in Figure 4. While all three primer sets exhibited strong 3′ end conservation, variations were observed in the 5′ end for some taxa, particularly *Bacteroides*, *Methanobrevibacter*, and *Roseburia*.

**Table 1:** The quantity of 16S rRNA gene sequences^a^ used in this study.

Gut microbiome genus	NCBI		SILVA	
	QC passed sequence	Length (bp)	QC passed sequence	Length (bp)
*Bifidobacterium*	100	1,462–1,576	100	1,506–1,553
*Collinsella*	100	1,415–1,580	100	1,342–1,516
*Bacteroides*	100	1,399–1,525	100	1,385–1,544
*Prevotella*	100	1,456–1,533	100	1,393–1,532
*Alistipes*	100	1,415–1,773	100	1,371–1,527
*Parabacteroides*	100	1,345–1,780	100	1,351–1,540
*Lacticaseibacillus*	100	1,503–1,547	100	1,425–1,579
*Streptococcus*	100	1,373–1,380	100	1,400–1,562
*Clostridium*	100	1,453–1,530	100	1,343–1,523
*Eubacterium*	100	1,435–1,525	23	1,379–1,539
*Blautia*	100	1,380–1,576	100	1,375–1,544
*Dorea*	100	1,456–1,534	100	1,373–1,534
*Lachnospira*	31	1,428–1,542	5	1,453–1,529
*Roseburia*	100	1,432–1,536	100	1,286–1,523
*Faecalibacterium*	100	1,468–1,505	100	1,330–1,519
*Ruminococcus*	100	1,380–1,351	100	1,364–1,538
*Subdoligranulum*	6	1,330–1,524	100	1,339–1,519
*Megamonas*	21	1,454–1,549	41	1,396–1,544
*Megasphaera*	73	1,200–1,658	84	1,322–1,574
*Escherichia-Shigella*	100	1,530–1,547	100	1,522–1,559
Total	1,731		1,753	

a The 16S rRNA gene sequences downloaded from the NCBI and SILVA databases were subjected to quality filtering prior to multiple sequence alignment. Some genera had a limited number of sequences that met the quality criteria.

## Discussion

16S rRNA gene sequencing remains a cornerstone of bacterial and archaeal community research, yet inherent biases can compromise the accuracy of taxonomic identification and community composition estimates (Yarza et al., 2014; Clooney et al., 2016). This study provides specific evidence of these limitations within the context of the core human gut microbiome, focusing on primer performance and the impact of intergenomic variation.

Our systematic analysis demonstrates that even widely used “universal” primers, including full-length 16S rRNA gene primers and other common sets, often fail to cover the complete diversity of the core gut microbiome. Two main factors contribute to this shortfall: unexpected variability in supposedly conserved primer binding sites (including insertions and point mutations) and the traditional reliance on cultured bacterial isolates for primer development (Nearing et al., 2021; Wang and Qian, 2009). Because cultured isolates represent only a fraction of naturally occurring microbes, many taxa now detectable by modern sequencing remain overlooked (Mao et al., 2012; Baker et al., 2003; Almeida et al., 2019). The dramatic escalation in recognized bacterial taxa (Lloyd et al., 2018) highlights the need for improved primer design strategies and refined reference databases such as SILVA (see text footnote 3) to capture the breadth of microbial diversity more accurately.

We evaluated three underutilized candidate primer sets (V3_P3, V3_P7, and V4_P10) and found that each provides a promising balance between coverage and specificity across all taxa (Supplementary Figures S4–S6). This finding aligns with recommendations suggesting that targeting the V3–V5 regions may help reduce bacterial diversity overestimation, particularly for phyla with high 16S rRNA gene copy numbers (Sun et al., 2013; Martínez-Porchas et al., 2016). The higher coverage observed with these primers may contribute to more accurate microbial compositions analysis, even when targeting similar regions with different primer sequences.

For example, candidate V3_P3 (U341F/533R) demonstrated broader coverage than the commonly used V3_P6 (338F/806R) by capturing a wider range of genera, including *Prevotella*, *Eubacterium*, *Blautia*, *Roseburia*, *Alistipes*, and *Subdoligranulum*-genera that are often underestimated by other primer sets when compared to shallow shotgun metagenomic data (Xu et al., 2021). Additionally, our candidate primer V3_P7 (341B4F/806R2) has shown robust performance, achieved a high genus-level classification accuracy (99.93%) and reliably reflected microbial abundance in wastewater and rumen fluid samples (Lu et al., 2015; Pang et al., 2021).

Similarly, candidate V4_P10 (515F/1061R) has been supported by studies demonstrating its ability to achieve highly accurate bacterial diversity profiling in mock community DNA while minimizing false positives (Winand et al., 2019). In contrast, commercially available V3–V4 primers are widely used despite evidence suggesting they may underrepresent specific bacterial taxa, as observed in previous studies. For example, Ning et al. (2022), using the commonly employed V3_P5 primer set targeting the V3–V4 region, reported differences in gut microbiome composition between healthy individuals and those with osteoarthritis but failed to detect the expected increase in *Alistipes*. Similarly, Talukdar et al. (2021), also using this primer set in diabetes research, occasionally failed to detect positive correlations between *Blautia* or *Roseburia* abundance and diabetes, as typically observed in shotgun metagenomic studies.

These discrepancies are likely due, in part, to mismatches between primer sequences and template DNA, as illustrated in Supplementary Figure S7. Beyond primer coverage, it is important to note that even a single mismatch within the last 3–4 nucleotides at the 3′ end of a primer can significantly reduce PCR amplification efficiency, even under optimal annealing temperatures (Mao et al., 2012). Our primer coverage analysis aligns with and extends previous reports regarding the underrepresentation of key gut microbiome taxa. Notably, our results corroborate earlier reports by Alcon-Giner et al. (2017) and Kameoka et al. (2021) demonstrating the underestimation of *Bifidobacterium* when using V1–V2 primers. We provide additional evidence for this, discrepancy through our *in silico* analysis, which reveals specific mismatches between V1–V2 primers and *Bifidobacterium* 16S rRNA sequences, as illustrated in Supplementary Figure S8. This observation underscores the importance of careful primer selection, particularly when targeting genera known to be critical in gut microbiome studies.

While our *in-silico* primer analysis suggests balanced coverage across core gut microbiome genera, it is important to acknowledge its dependence on available 16S rRNA sequences, which remain limited for certain genera. Consequently, taxa with low sequence representation may be underrepresented or entirely excluded in experimental studies, potentially skewing diversity estimates.

Additionally, our primers target only a partial 16S rRNA region (V3–V5), which may constrain taxonomic resolution. This limitation is particularly evident for closely related species or those with highly conserved sequences within this region, such as *Lachnospira*, *Faecalibacterium*, and *Escherichia-Shigella*. Recent studies have highlighted the challenges in distinguishing between these taxa using partial 16S sequencing alone.

The choice between partial and full-length 16S rRNA sequencing, often influenced by primer selection, significantly impacts the achievable level of taxonomic resolution. Researchers must carefully consider their specific objectives when selecting sequencing approaches. While partial sequencing is optimal for general diversity profiling and community-level analysis, full-length sequencing provides greater taxonomic resolution and improved species-level classification (Pan et al., 2023; Johnson et al., 2019; Buetas et al., 2024). This trade-off between broad coverage and detailed resolution underscores the importance of aligning sequencing strategy with research goals.

To mitigate these limitations, future studies could benefit from combining our proposed primer sets with complementary approaches, such as shotgun metagenomics or targeted full-length 16S sequencing for taxa of particular interest. This multi-faceted approach would provide a more comprehensive and accurate representation of the gut microbiome.

Our findings extend beyond the limitations of individual primer sets, revealing the complex interplay between the intergenomic variation and primer design in 16S rRNA gene sequencing. The substantial diversity observed within traditionally “conserved” regions highlights the dynamic nature of bacterial genomes, shaped by host-specific factors and environmental pressures (Kurilshikov et al., 2017). This variability challenges the concept of truly universal primer binding sites and highlights the need for adaptive strategies in microbiome research.

Building on these observations, we propose that a strategic combination of complementary primer sets could significantly improve the breadth and accuracy of microbial community profiling. This approach leverages the strengths of existing commercial primers that target overlapping regions, potentially offering a more comprehensive view of microbial diversity while mitigating individual primer biases. Such a multi-primer strategy aligns with the growing recognition of the gut microbiome’s complexity and the need for more nuanced analytical approaches in microbiome studies.

Database choice represents a critical factor in microbiome research, as highlighted by our findings. Discrepancies between expected intergenomic patterns and those observed in widely used repositories like NCBI and SILVA can skew taxonomic classification. This is exemplified by the unexpected variation we found in the traditionally “conserved” region of *Bifidobacterium*. Our analysis shows the importance of using updated, curated databases with robust coverage of uncultured organisms for accurate interpretation of amplicon data, especially in gut microbiome studies. We recommend employing multiple databases in parallel to strengthen analyses, as this approach can fill in gaps, reduce classification errors, and provide a more complete picture of microbial diversity. For instance, our comparison of NCBI and SILVA databases revealed complementary strengths, with each capturing unique aspects of 16S rRNA gene variation across different genera. This multi-database strategy aligns with recent trends in microbiome research, such as the development of specialized databases like MIMt (Cabezas et al., 2024) and GTDB (Parks et al., 2022), which offer improved taxonomic resolution and representation of uncultured microorganisms.

Although 16S rRNA gene sequencing provides a broad overview of bacterial composition, it may not fully capture the absolute abundance or diversity of individual taxa. For reliable comparisons across studies, it is crucial to employ consistent 16S rRNA gene sequencing methodologies, thereby minimizing biases introduced by different primer sets or analytical pipelines. Moreover, aligning 16S rRNA gene data with shotgun metagenomic data poses additional challenges due to differences in sequencing depth and data processing techniques. Researchers should be aware of these limitations and interpret results cautiously, particularly when comparing data from different studies or methodologies.

Our approach identifies candidate primer sets that maximize the sensitivity of 16S rRNA gene amplification, enabling accurate capture of bacterial profiles across diverse conditions. This enhanced sensitivity facilitates the detection of microbiome changes associated with various health conditions and applications beyond human gut microbiome studies.

In the context of human health, these primers could improve detection of microbiome alterations related to Inflammatory Bowel Disease, including the underrepresentation of genera such as *Lactobacillus*, *Bifidobacterium*, and *Faecalibacterium* (Zuo and Ng, 2018). They may also prove valuable in studies examining the reduction of butyrate-producing bacteria, such as *Faecalibacterium prausnitzii* and *Roseburia intestinalis*, which are linked to the onset and progression of type 2 diabetes in both animal and human studies (Crudele et al., 2023).

Given their demonstrated coverage of core gut microbiome genera, these candidate primers have potential applications in broader microbial ecology research. For instance, they could be applied to animal microbiome studies, particularly in poultry research focusing on the chicken gut microbiome and its impact on meat and egg production (Shang et al., 2018). Additionally, these primers may enhance probiotic community profiling in human wellness and fermented food research, where *Lacticaseibacillus*, *Streptococcus*, and other lactic acid bacteria play crucial roles (De Filippis et al., 2020). Furthermore, the improved sensitivity and coverage of these primers could benefit environmental microbiome studies, including soil and water ecosystem analyses, potentially revealing previously underrepresented microbial diversity in these complex environments. This broader applicability underscores the value of our findings beyond human gut microbiome research, offering tools for more comprehensive microbial community profiling across various fields of study.

While our *in silico* analyses highlight the potential of certain primer sets, we acknowledge that need for rigorous *in vitro* validation to confirm their effectiveness under real-world conditions. This limitation underscores the importance of bridging computational predictions with experimental data. Future research should integrate 16S rRNA intergenomic variation with curated primer databases to develop advanced computational tools for tailoring primer design to specific questions and taxa of interest.

Our study illuminates the complexities of 16S rRNA gene-based gut microbiome profiling by revealing the limitations of universally applied primers and emphasizing the critical roles of intergenomic variation and database selection in shaping research outcomes. By comparing our findings with studies in diverse environments such as soil, water, and fermented foods, we demonstrate the broader applicability of our approach beyond gut microbiome research. This comparative perspective enhances the value of our findings and underscores the need for careful primer selection across various microbiome studies.

Furthermore, our work aligns with and extends previous research on the overestimation of prokaryotic diversity, reinforcing the importance of considering both inter- and intragenomic variation in 16S rRNA genes when designing primers and interpreting sequencing results (Martínez-Porchas et al., 2016). These insights can guide the development of more precise, reliable, and reproducible methodologies in microbiome studies across various fields.

In conclusion, this study not only advances our understanding of the intricate relationships between host health and gut microbial communities but also provides a framework for improving microbiome research methodologies more broadly. By addressing the challenges in primer design and emphasizing the need for tailored approaches, our work contributes to the ongoing refinement of tools and strategies for exploring the vast and complex world of microbial ecology.

## Data Availability

The datasets presented in this study can be found in online repositories. The names of the repository/repositories and accession number(s) can be found in the article/Supplementary material.

## Footnotes

^1^ https://pubmed.ncbi.nlm.nih.gov

^2^ http://omegabioservices.com/index.php/16s-reference/

^3^ https://www.arb-silva.de/

^4^ https://weblogo.threeplusone.com/create.cgi

^5^ https://www.ncbi.nlm.nih.gov/nucleotide/

^6^ https://www.hiv.lanl.gov/content/sequence/ENTROPY/entropy_one.html
