# Supplementary figures and images for: Unveiling the impact of 16S rRNA gene intergenomic variation on primer design and gut microbiome profiling
Source: Front Microbiol. 2025 May 2;16:1573920. doi: 10.3389/fmicb.2025.1573920 (PMC12081361; doi:10.3389/fmicb.2025.1573920)

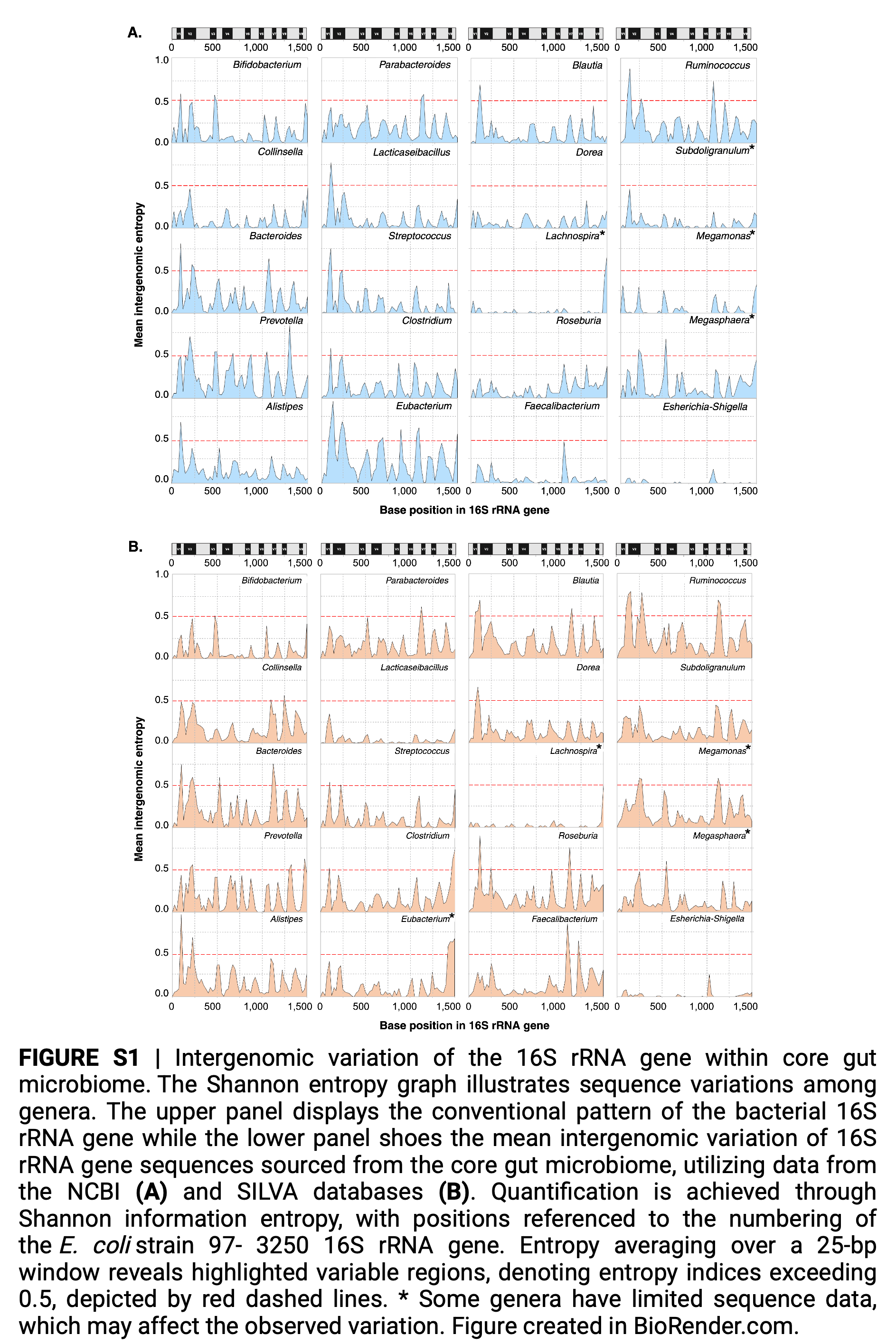

Supplement: Supplementary file 1 [file Image_1.TIFF]

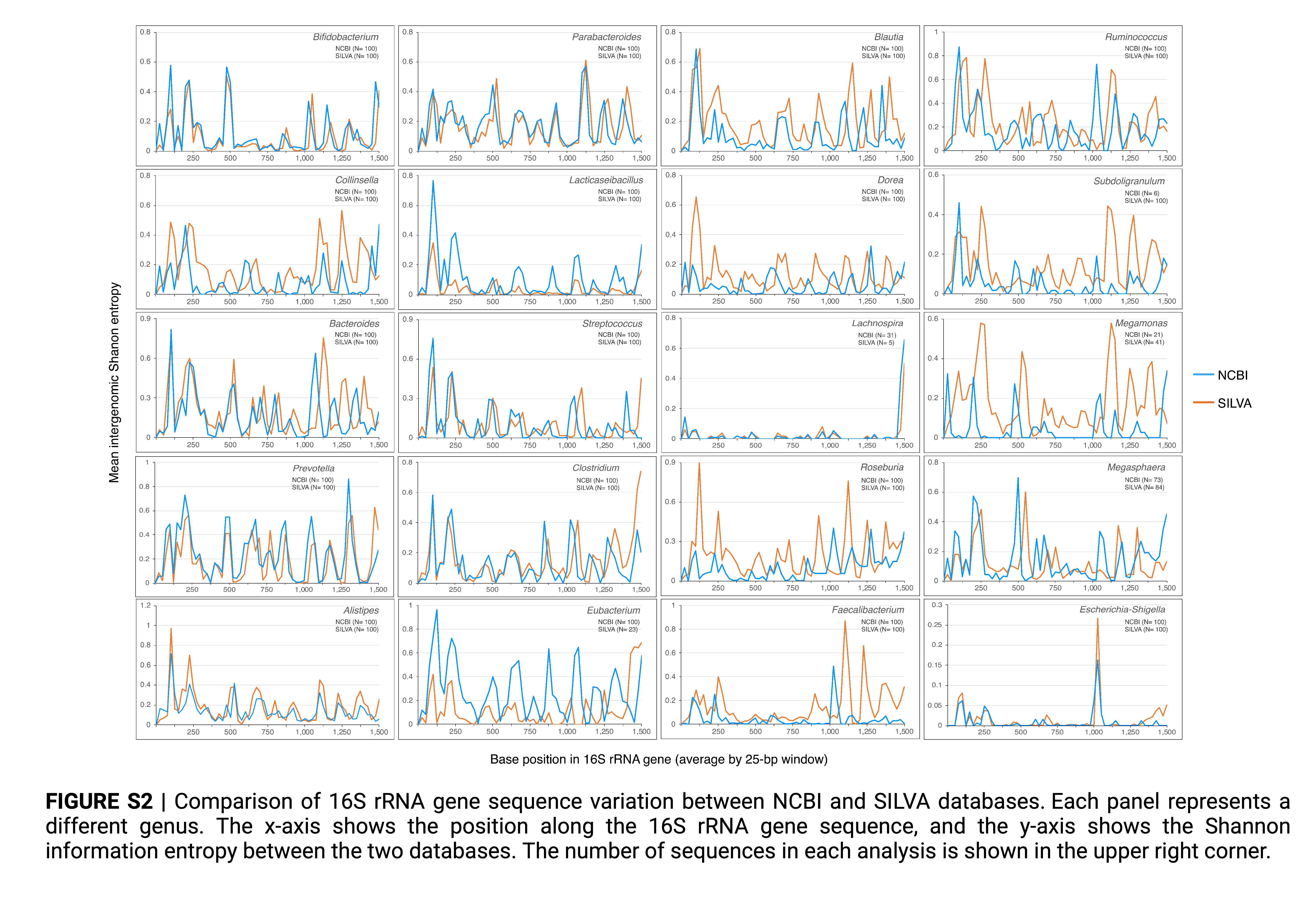

Supplement: Supplementary file 2 [file Image_2.TIFF]

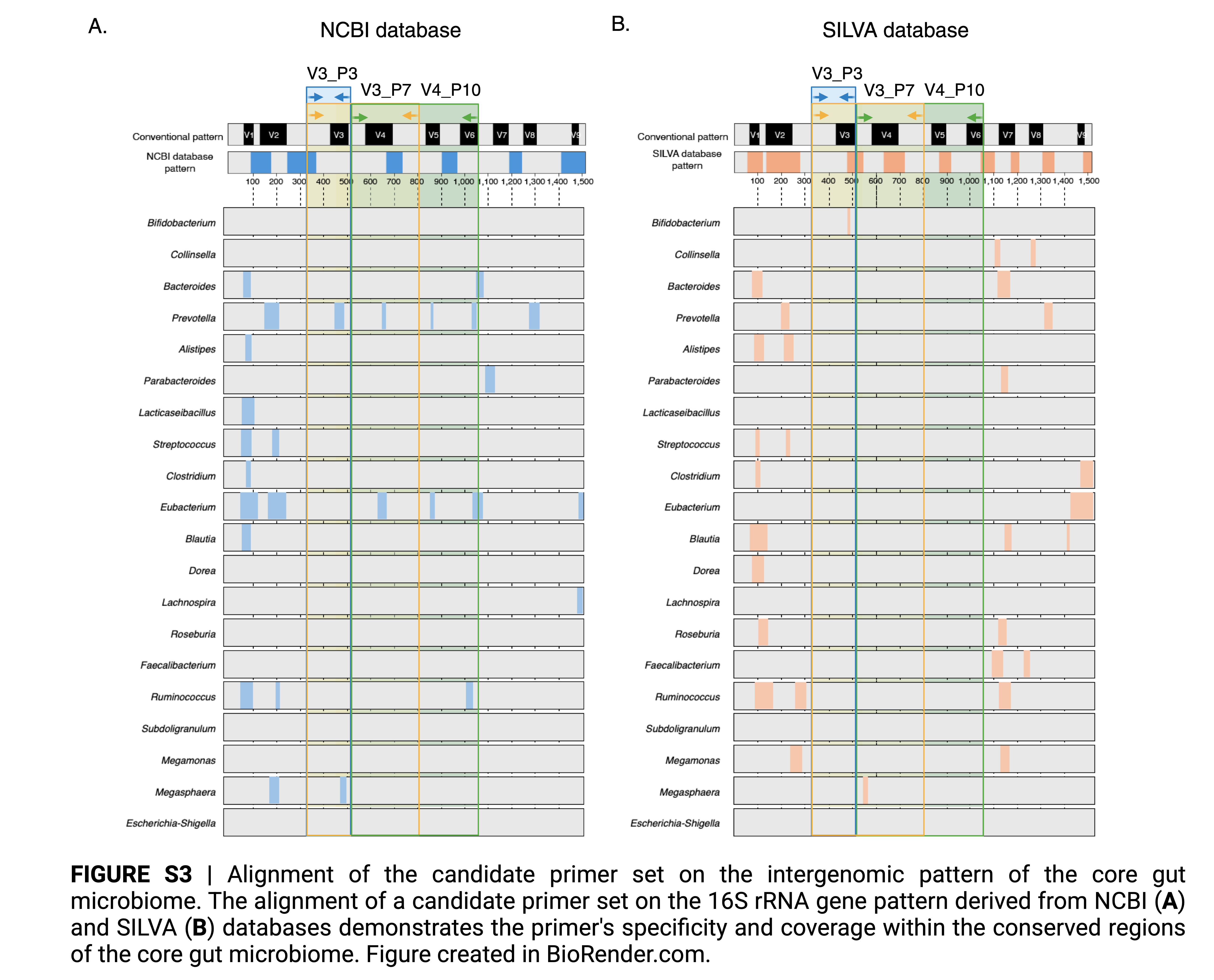

Supplement: Supplementary file 3 [file Image_3.TIFF]

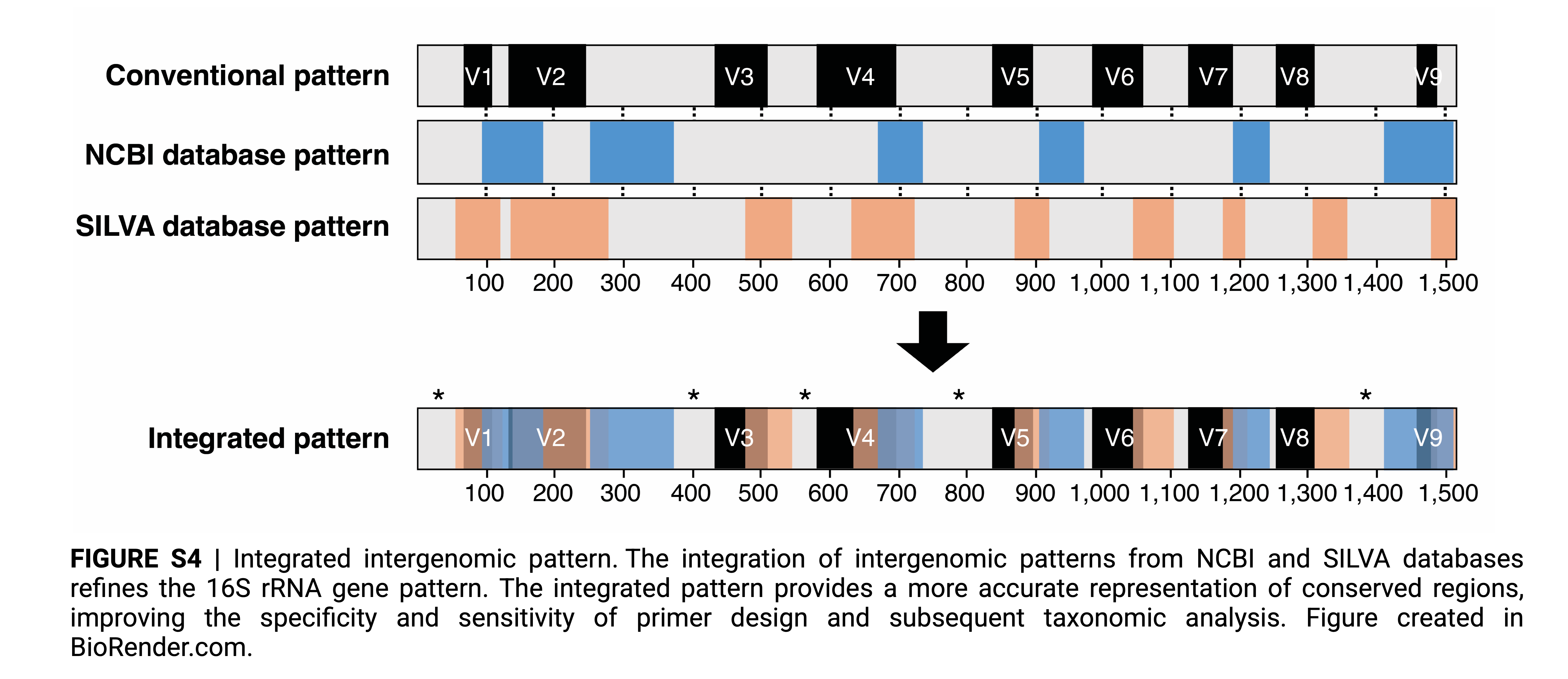

Supplement: Supplementary file 4 [file Image_4.TIFF]

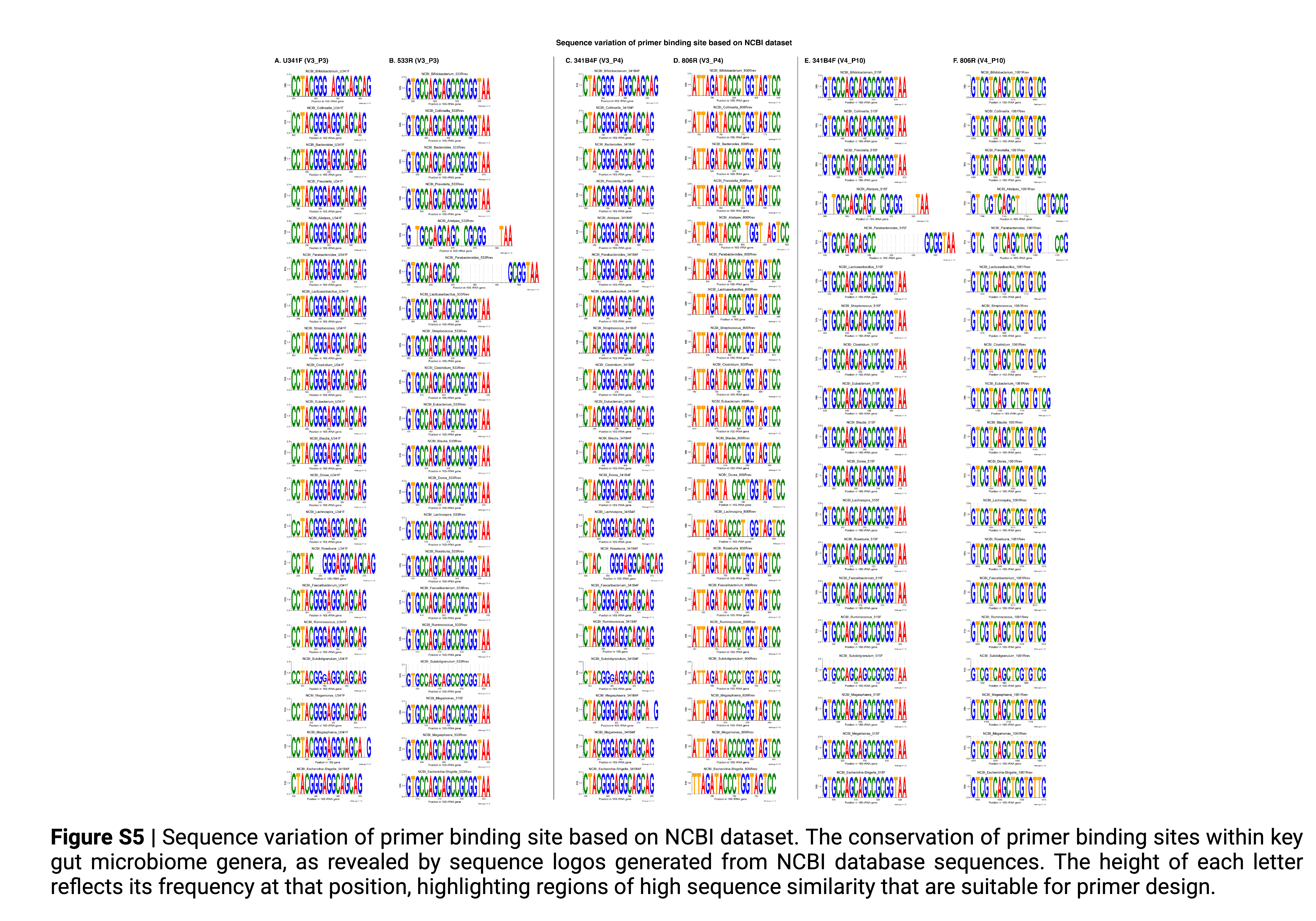

Supplement: Supplementary file 5 [file Image_5.TIFF]

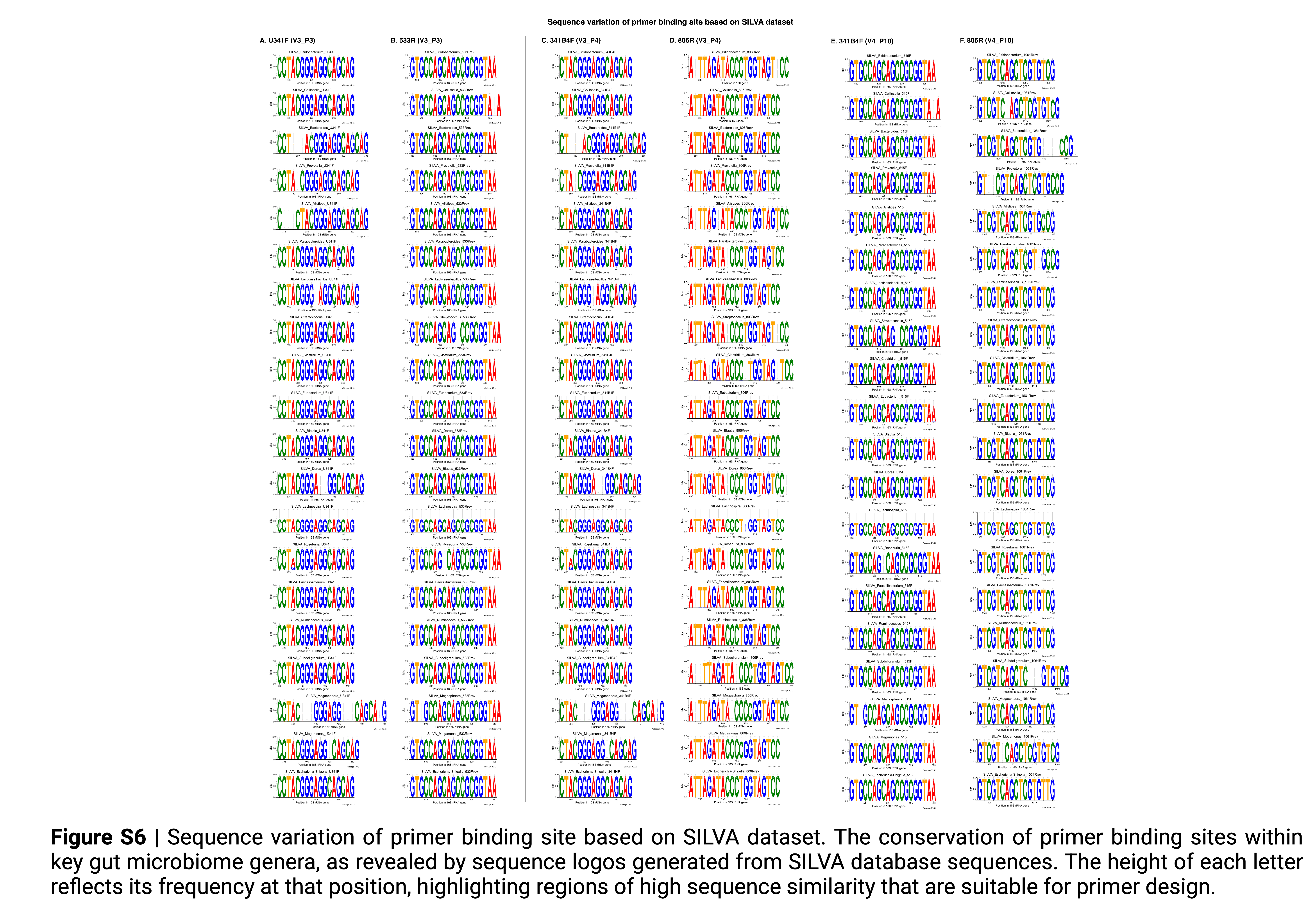

Supplement: Supplementary file 6 [file Image_6.TIFF]

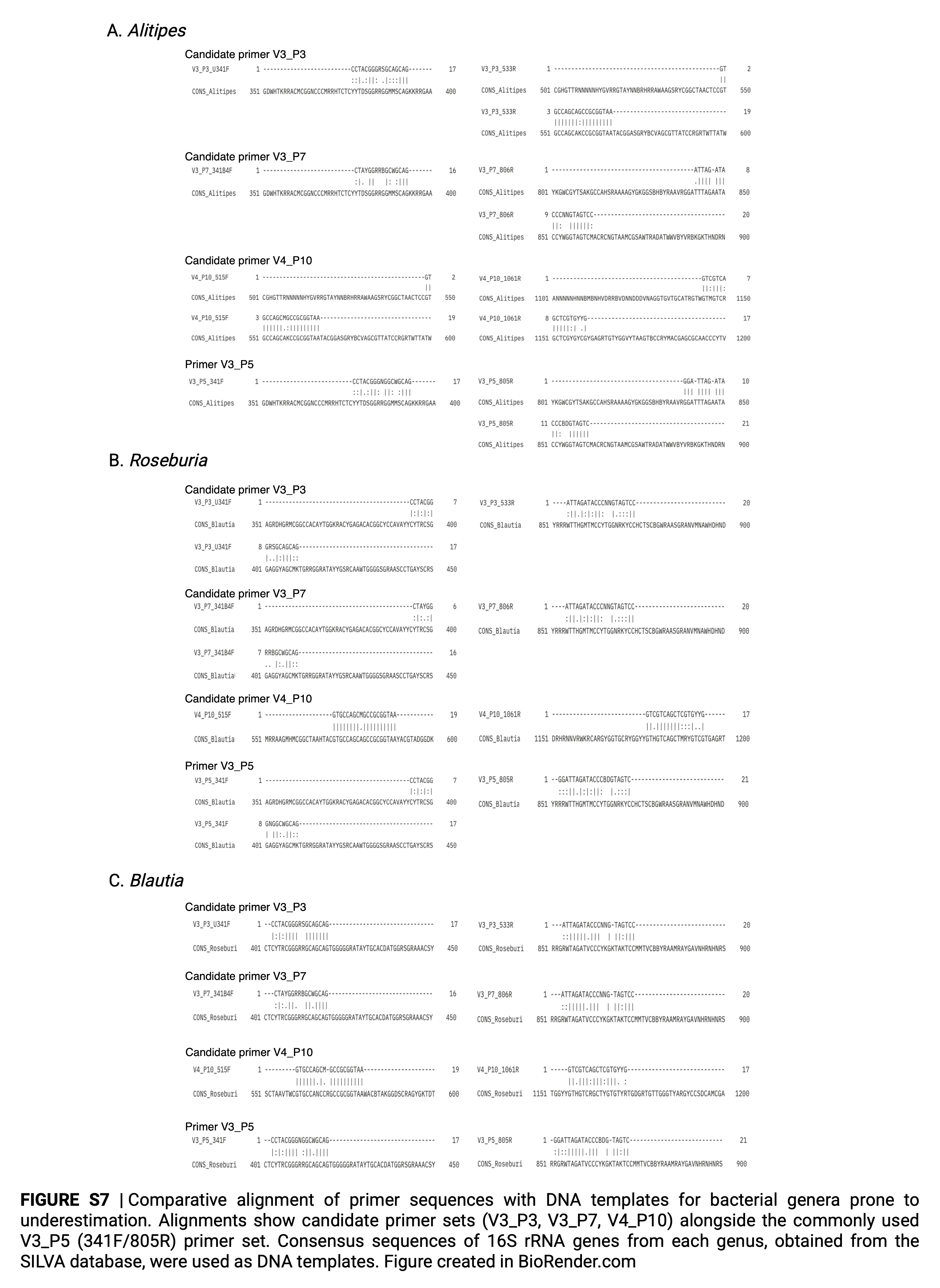

Supplement: Supplementary file 7 [file Image_7.TIFF]

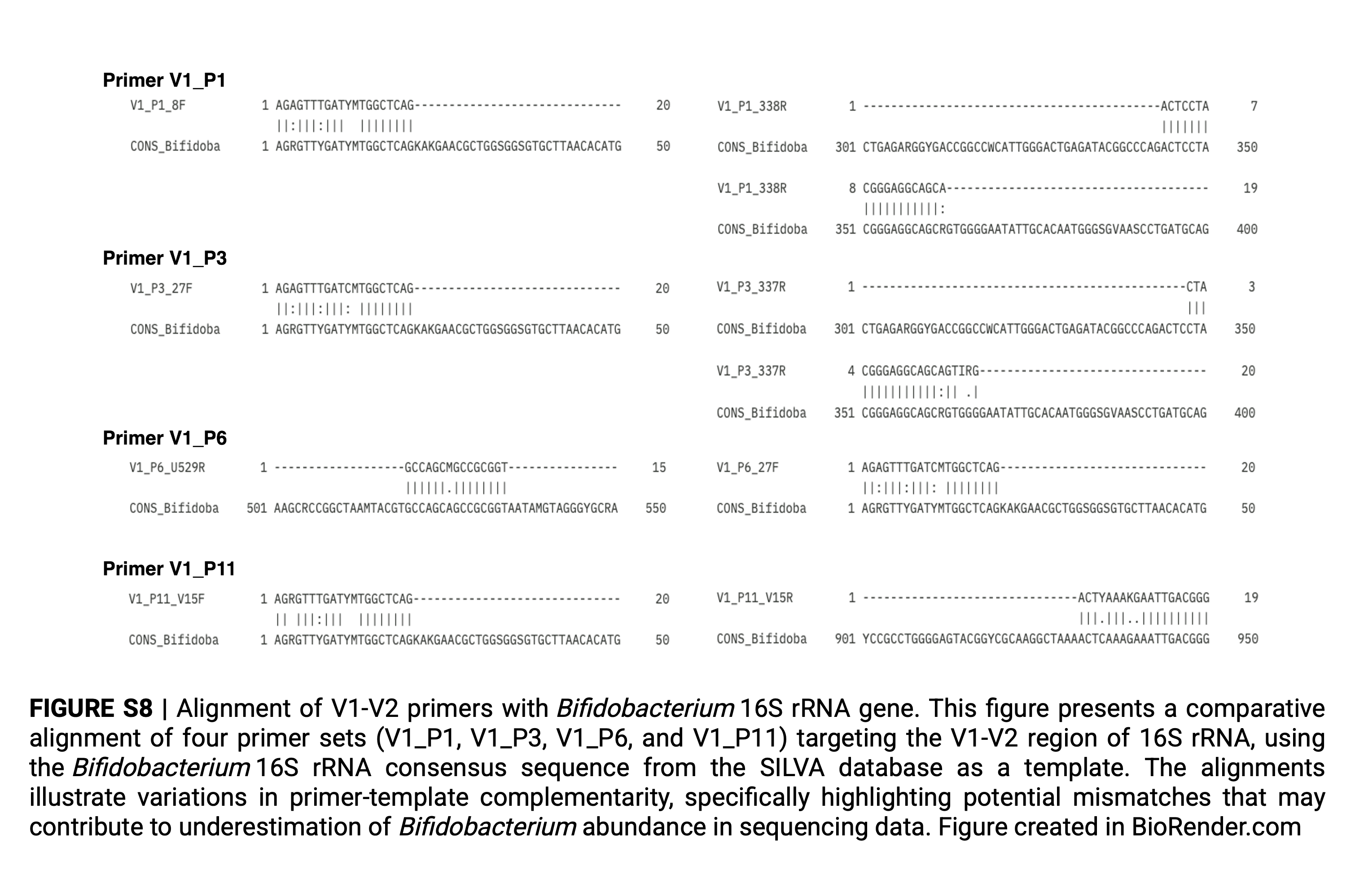

Supplement: Supplementary file 8 [file Image_8.TIFF]
